# Investigation of Potent Lead for Acquired Immunodeficiency Syndrome from Traditional Chinese Medicine

**DOI:** 10.1155/2014/205890

**Published:** 2014-06-12

**Authors:** Tzu-Chieh Hung, Wen-Yuan Lee, Kuen-Bao Chen, Yueh-Chiu Chan, Calvin Yu-Chian Chen

**Affiliations:** ^1^Department of Biomedical Informatics, Asia University, Taichung 41354, Taiwan; ^2^School of Medicine, College of Medicine, China Medical University, Taichung 40402, Taiwan; ^3^Department of Neurosurgery, China Medical University Hospital, No. 2, Yude Road, North District, Taichung City 40447, Taiwan; ^4^Department of Anesthesiology, China Medical University Hospital, Taichung 40447, Taiwan; ^5^Research Center for Chinese Medicine & Acupuncture, China Medical University, Taichung 40402, Taiwan

## Abstract

Acquired immunodeficiency syndrome (AIDS), caused by human immunodeficiency virus (HIV), has become, because of the rapid spread of the disease, a serious global problem and cannot be treated. Recent studies indicate that VIF is a protein of HIV to prevent all of human immunity to attack HIV. Molecular compounds of traditional Chinese medicine (TCM) database filtered through molecular docking and molecular dynamics simulations to inhibit VIF can protect against HIV. Glutamic acid, plantagoguanidinic acid, and Aurantiamide acetate based docking score higher with other TCM compounds selected. Molecular dynamics are useful for analysis and detection ligand interactions. According to the docking position, hydrophobic interactions, hydrogen bonding changes, and structure variation, the study try to select the efficacy of traditional Chinese medicine compound Aurantiamide acetate is better than the other for protein-ligand interactions to maintain the protein composition, based on changes in the structure.

## 1. Introduction

Human immunodeficiency virus (HIV) is a retrovirus that causes acquired immunodeficiency syndrome (AIDS) [1–4]. In the immune system, AIDS is caused by a virus and then allowed opportunistic infections and cancers, damage to flourish. Unprotected sexual intercourse [5, 6], contaminated medical devices (blood transfusions, surgery, and sharing needles) [7, 8], vertical transmission (pregnancy, childbirth, or breastfeeding) [9, 10], and body fluids make virus be transmitted through a population rapidly.

There were 35.3 million people living with HIV in 2012 (recorded by WHO). There are still no defined vaccines or drugs approval to kill all HIV virus in patient. The highly active antiretroviral therapy (HAART) is the standard of care for patients with advanced infection in current treatment [11]. HARRT is using a complex of transcription inhibitors to slow down transcription and then make the patient's total burden of HIV decrease, but this treatment is too expensive.

Recent studies indicate that viral infectivity factor (VIF) is an important goal of AIDS [12] in 2014. VIF is a protein in a lot retrovirus to degrade human enzyme APOBEC which can break down the unprotected virus. The virus can exist in human with VIF, thus the inhibition of VIF could help the immunity system to kill the virus.

Computer-aided drug design (CADD) is a technique for drug design based on computer simulation. The difference from traditional drug design is that CADD has the advantages of higher speed and lower cost to the screening of new compounds by the structure and biological activity of control, that is, two main applications named structure based and ligand based drug design of computer-aided drug design [13–18]. In this research, we use computer-aided drug design, molecular modeling in drug design basics to focus on drug design and molecular structure dynamics.

The personalized medicine and biomedicine are famous knowledge in these years. On the analysis of regional diseases [19], rare diseases [20], clinical diagnosis cases [21, 22], and disease associated mutations [23–25], this knowledge has drawn more and more attention [26, 27]. Traditional Chinese medicine (TCM) is defined as a personalized medicine that has long been an important culture in Asia. The TCM Database@Taiwan (http://tcm.cmu.edu.tw/) [28] is the largest traditional Chinese medicine database in the world which has been established in 2011. This database has 2D chemical structure and 3D chemical structure, and the bioactivity of 61,000 compounds extracted from TCM herbs can be searched. Since 2011, the TCM Database@Taiwan application has been investigated for treatments of insomnia [29], pigmentary disorders [30], Parkinson's disease prevention [31], EGFR inhibition [32], pain relief [14], and antivirals [33–37]. Recently, the TCM Database@Taiwan is helpful to screen TCM compounds via a cloud computing platform [38, 39].

In this research, we select TCM compounds to inhibit VIF by analyzing their interactions. The candidate compounds are selected based on the docking and structure variations and analyzed the interaction through molecular simulation.

## 2. Materials and Methods

### 2.1. Data Set

The traditional Chinese medicine compounds could be downloaded from the database (http://tcm.cmu.edu.tw/) and generate the small molecule compounds to identify potential VIF agonist screening.

The VIF protein sequences from UNIPROT acquired knowledge (P12504, HIV) and a three-dimensional structure of VIF protein could be offered from the Protein Data Bank (PDB ID: 4N9F) [12].

### 2.2. Structure Based Virtual Screening

The TCM compounds docking to VIF is performed by using LigandFit module in DS 2.5 [40]. Under Harvard molecular mechanics force field (CHARMM) [41], all docking posture chemistry is minimized. We make the DS 2.5 LigandFit module calculate piecewise linear potentiometer (-PLP) score and docking score. LIGPLUS [42, 43] calculates hydrogen bonding and hydrophobic contacts (hydrogen) during the ligand and protein interaction.

### 2.3. Disorder Prediction

We use PONDR-FIT program DisProt [44] exclusion VIF receptor site to define the character of three-dimensional structure. The comparison between disorder region and docking site could help the definition of drug efficacy.

### 2.4. Molecular Dynamics (MD) Simulations

We used chemical simulation package Groningen machine (GROMACS 4.5.5) to molecular dynamics simulations [45]. Before MD, these selected ligands must be prepared by using SwissParam (http://swissparam.ch/) [46] on the force field [47]. The complex is transferred to the buffer (or solution) simulation box. The distance between the complex and the box is 1.2 Å. This TIP3P water-solution model contains sodium and chloride ions to neutralize complex charges full in box. The minimization used the steepest descent method for 5,000 steps; then the final structure with the lowest energy was transferred to MD simulation. The electrostatic interactions were calculated based on the particle mesh Ewald (PME) method with 2 fs per time step for a total of 5,000,000 iterations [48]. The equilibration was under a 100 ps constant temperature (NVT ensemble) based on the Berendsen weak thermal coupling method. The minimum distance of the root locus analysis of digital root mean square deviation (RMSD), total energy, RMS fluctuations (RMSF), residue matrix database structure assignment (DSSP), and cluster analysis could be calculated from the program of MD simulations.

### 2.5. FasL Pathway

We use the software caver 3.0 to analyze all possible ways interpath protein path during MD simulation [49].

## 3. Results and Discussion

### 3.1. Disorder Prediction

**Figure 1:**
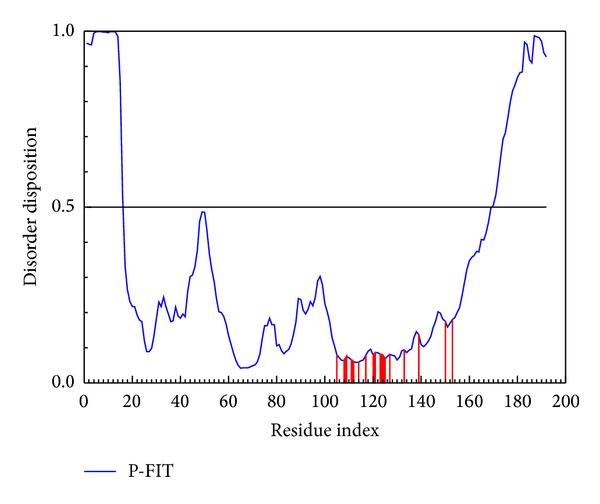
The disorder and binding site detection. The blue curve in the figure is the disorder disposition of each amino acid, and the red lines present the residues of the important amino acids for docking site.

Protein disorder is defined as an unstructured protein, and such characters for the docking site will make drug docking to the protein as complex difficultly. The references [50, 51] present that protein disorder is not a common domain; thus, the drug might have less side effects during the interaction. For the above reasons, disorder for drug design is not a bad situation, and it should be defined as difficult work only. The important amino acids of VIF Gln105, His108, Leu109, Tyr111, Phe112, Cys114, Glu117, Ile120, Arg121, Thr123, Ile124, Leu125, Arg127, Cys133, His139, Leu150, and Ile153 are defined as nondisorder regions (Figure 1). For the result, the selected compound dock to VIF will not be difficult.

### 3.2. Docking

The top three TCM compounds can be selected from the database according to the rank of the result of molecular docking by docking score (Table 1). These TCM compounds are glutamic acid, plantagoguanidinic acid, and Aurantiamide acetate extract from the TCM herbs* Achyranthes bidentata Bl.*,* Plantago asiatica L.*, and* Cordyceps sinensis *(*Berk*.)* Sacc *(or* Sargassum pallidum *(*Turn*.) and* Lycium chinense Miller*). The top compound, glutamic acid, is extracted from* Achyranthes bidentata Bl.* with the function to develop immunity [52, 53]. The second ranked herb, plantagoguanidinic acid, with the herb* Plantago asiatica* L. had been defined owing to the function of antiviral and immunomodulatory [54, 55]. The third ranked compound,* Aurantiamide acetate,* could be extracted from the herb* Sargassum pallidum *(*Turn.*)*, Cordyceps sinensis *(*Berk.*)* Sacc., *and* Lycium chinense *Miller. These herbs have recorded the function to improve the immunity [56–62]. From these references, we suggest our selected compounds might inhibit VIF from the immunity regulation.

**Figure 2:**
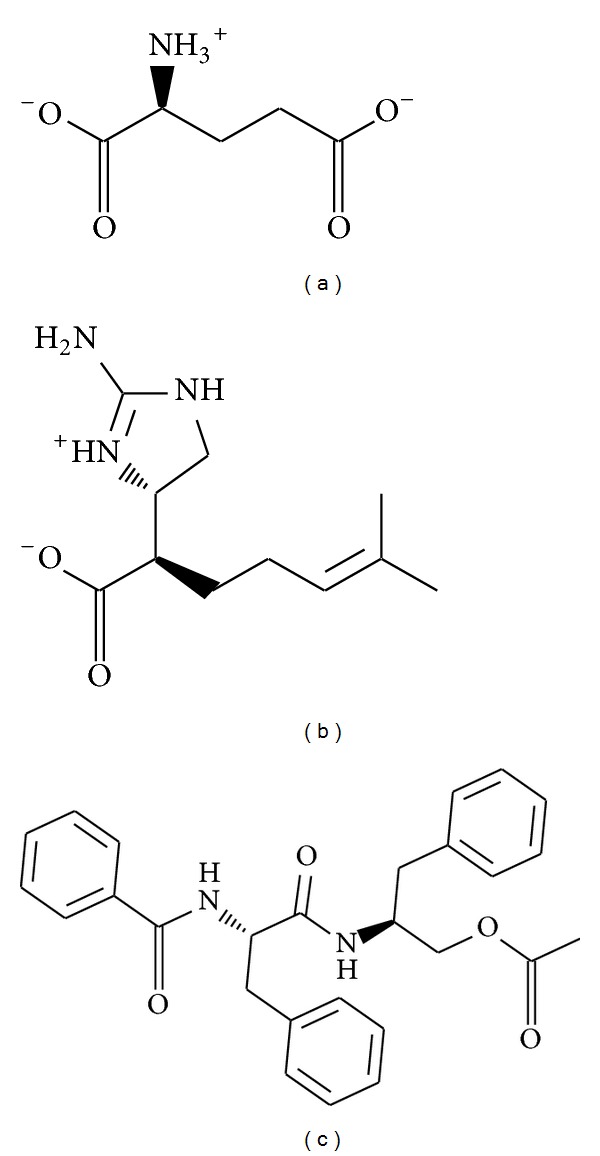
The structure of control and candidate TCM compounds. (a) Glutamic acid, (b) plantagoguanidinic acid, and (c) Aurantiamide acetate.

**Figure 3:**
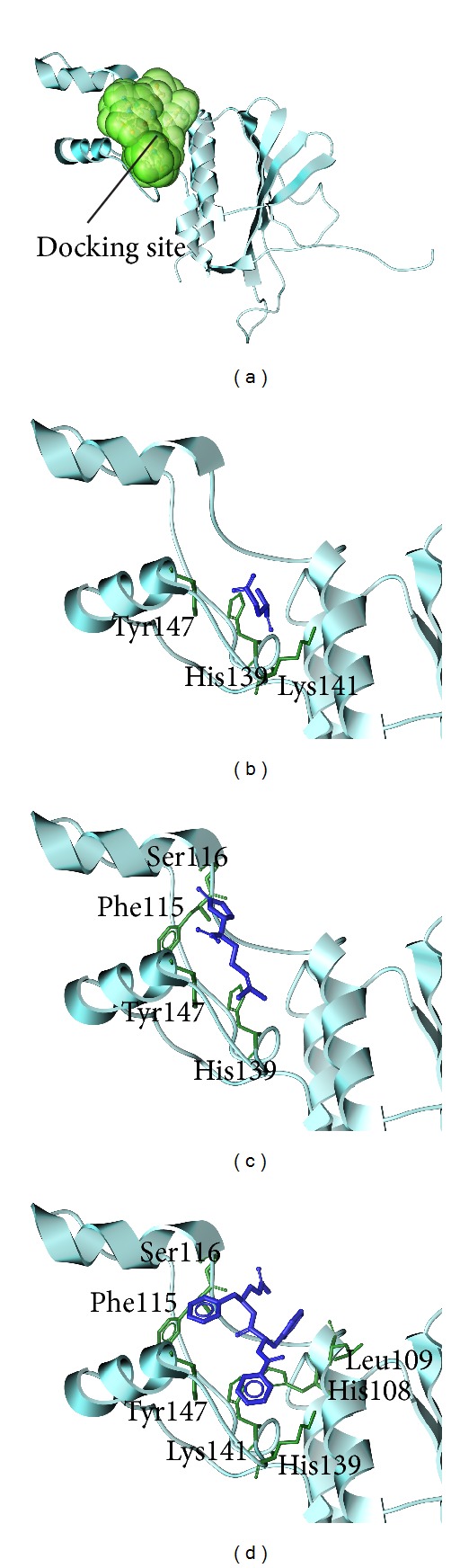
The docking poses of ligands. (a) The crystal structure of VIF and the designed docking site, (b) glutamic acid, (c) plantagoguanidinic acid, and (d) Aurantiamide acetate.

The structure of the candidate compounds selected after screening is shown in (Figure 2). The docking poses show the docking site and the important amino acid near ligands (Figure 3). From this result, we observe some amino acids may play important roles in a VIF target function.

**Figure 4:**
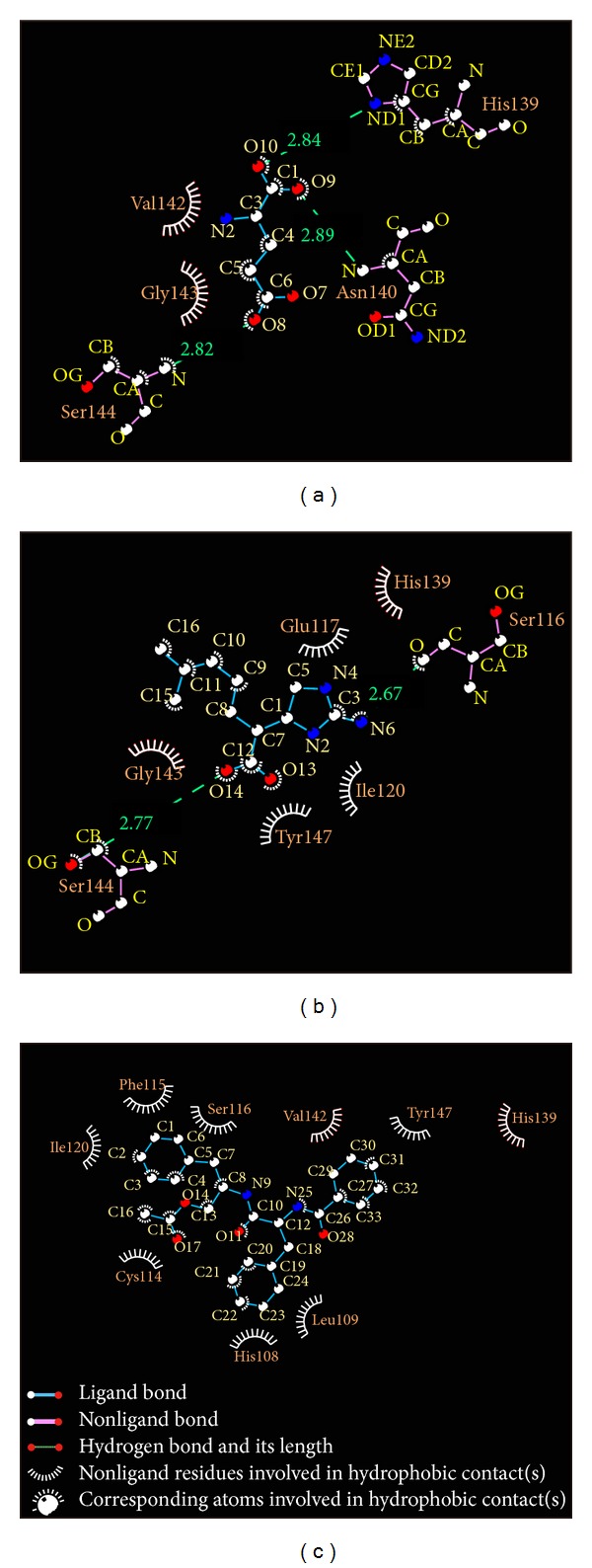
Ligplot illustrates the protein-ligand interactions. (a) Glutamic acid, (b) plantagoguanidinic acid, and (c) Aurantiamide acetate. The light red color of the hydrophobic interactions indicates a high frequency in all ligand interactions.

The hydrophobic interaction can be analyzed by LIGPLUS (Figure 4). This result shows that the amino acids His139 can have interactions with the ligands through hydrophobic interactions or hydrogen bonds that might be as important as amino while the selected compounds have an effect on VIF.

### 3.3. Molecular Simulation

**Figure 5:**
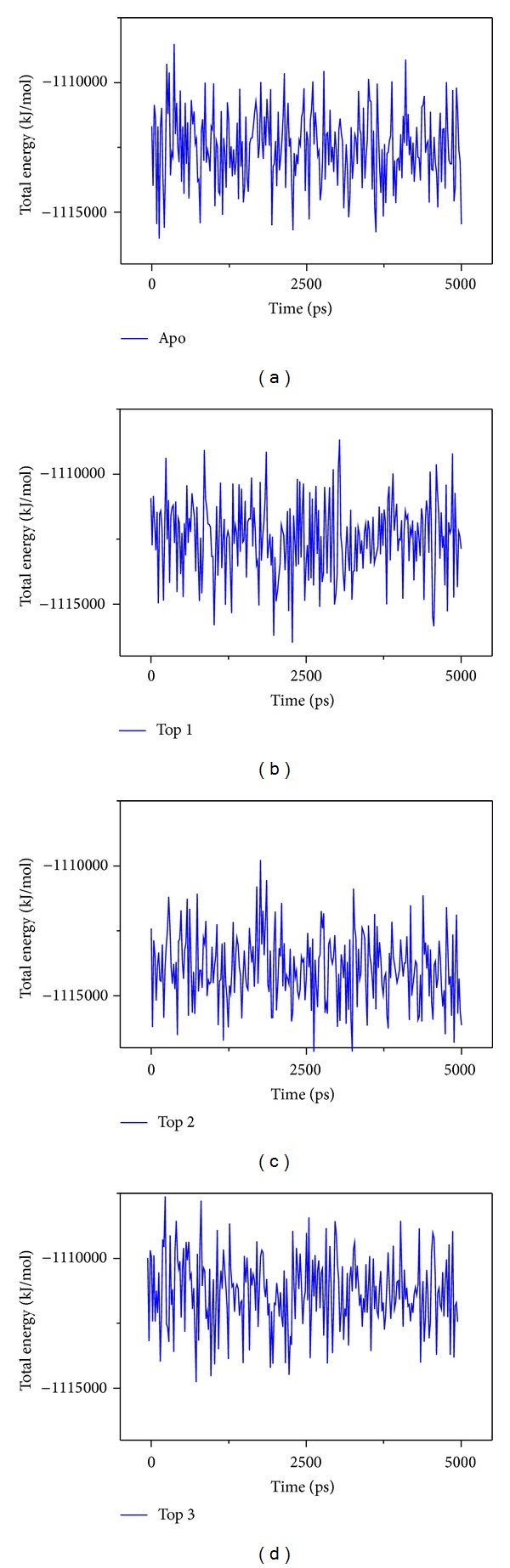
Measuring the energy variation of the complex. (a) Apo VIF, (b) glutamic acid, (c) plantagoguanidinic acid, (d) Aurantiamide acetate.

**Figure 6:**
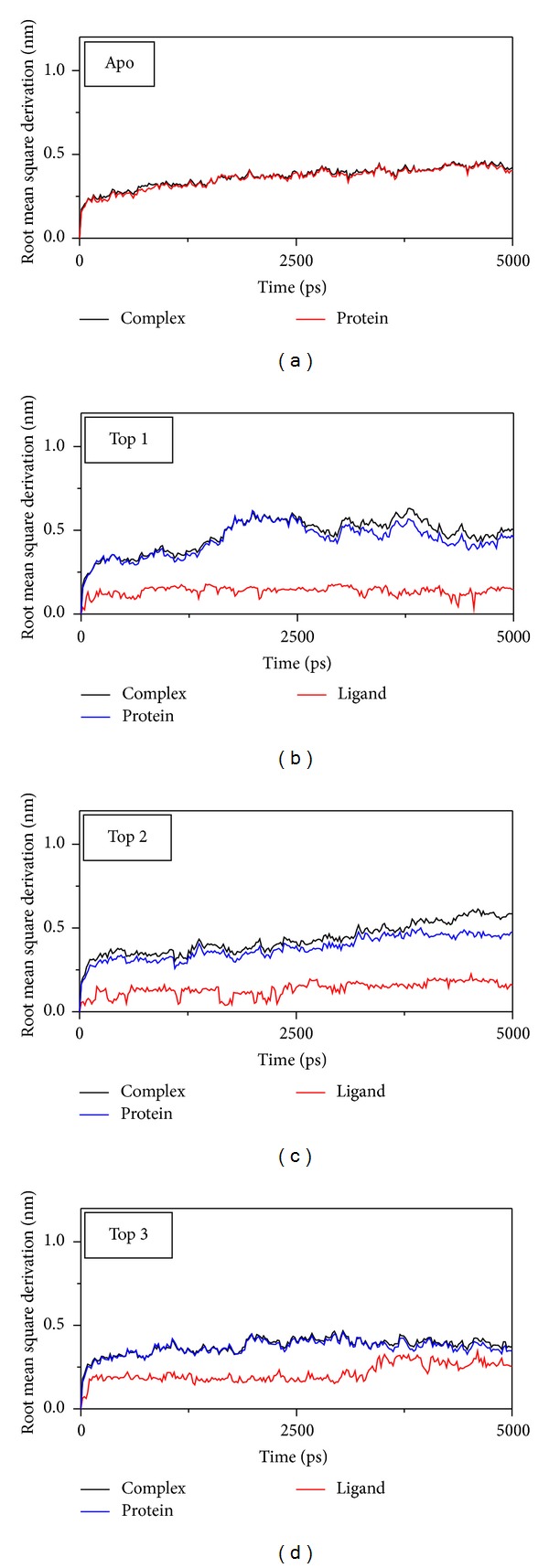
Measures the RMSD variation of the complex. (a) Apo VIF, (b) glutamic acid, (c) plantagoguanidinic acid, and (d) Aurantiamide acetate.

**Figure 7:**
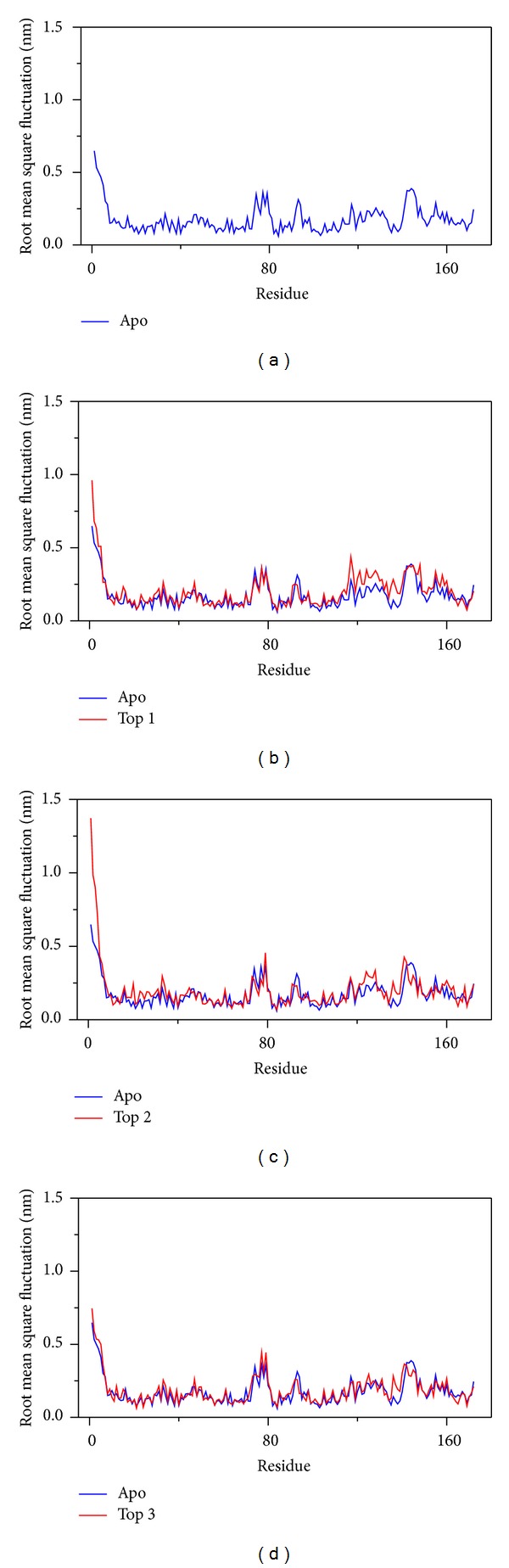
The variation of RMSD focuses on residue of protein and compares with apo (unbound protein). (a) Apo VIF and apo compare with (b) glutamic acid, (c) plantagoguanidinic acid, and (d) Aurantiamide acetate.

The RMSD and total energy of a complex during MD simulation were recorded (Figures 5–7). The total energy is in the range of −111.5~−111∗10^3^ kJ/mol. The amplitude is gentle; then, we suggest the interaction for VIF and compounds tend to balance (Figure 5). In these compounds, the top 2 plantagoguanidinic acid has the lowest energy that means the complex might be the most stable. The complex RMSD in top 1 and top 2 is different from protein, which might mean that ligand moves away from the docking site (Figure 6). In Figure 6, we also find that the top 3 Aurantiamide acetates have the lowest RMSD among others (contain apo/unbound protein). The variation of ligand is continue might present Aurantiamide acetate interaction with VIF will make the complex stable.

The RMSD focus on each residue (means RMSF) could detect the variation of protein during interaction (Figure 7). In this result, we can find that the pick sites of protein with ligand interaction are similar to apo protein and the value is larger than apo protein, which means the docking site is designed as the functional site and ligands interactions affect these residues by different force.

**Figure 8:**
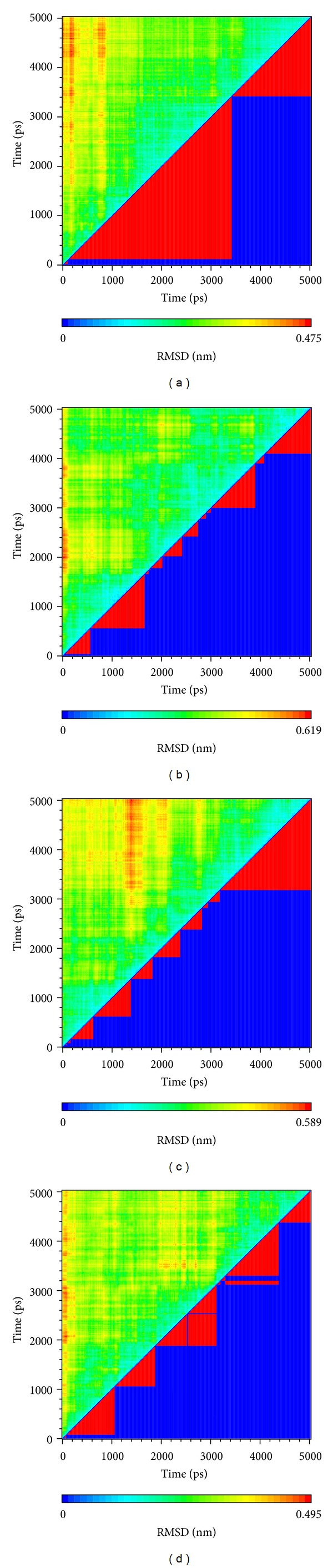
The clustering of the ligand-protein interaction. (a) Apo VIF, (b) glutamic acid, (c) plantagoguanidinic acid, and (d) Aurantiamide acetate.

The clustering is based on RMSD variation to divide data into several groups. This clustering method could make the similar structure in the same group (Figure 8). In this figure, complex with ligands will have more groups than apo. It might be presented that the ligands target to VIF might make larger structure variation and then inhibit the function of protein.

**Figure 9:**
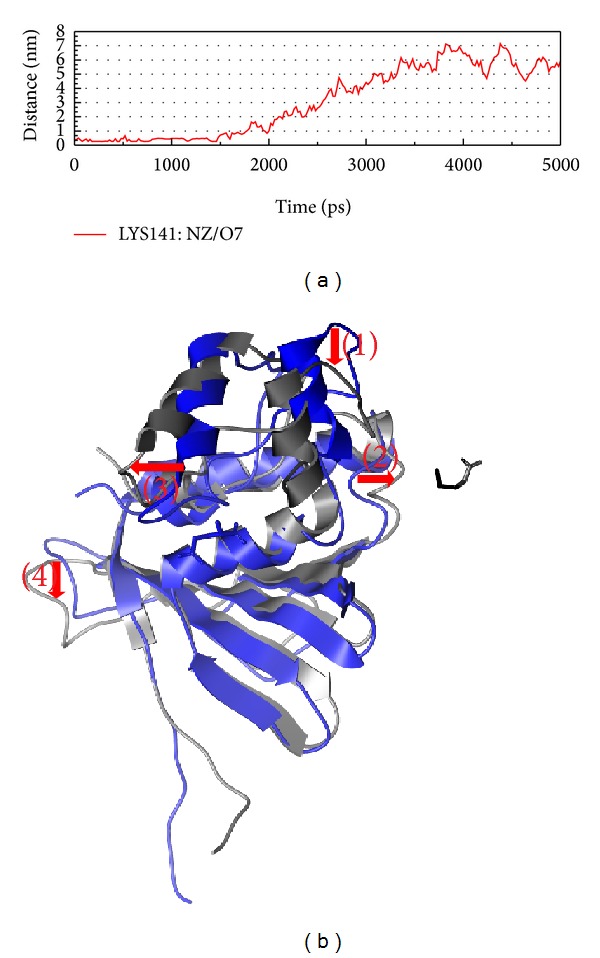
The variation of glutamic acid and VIF complex in MD simulation. (a) H-bond variation, (b) structure variation. The (1)–(4) red color indicates the difference through MD.

**Figure 10:**
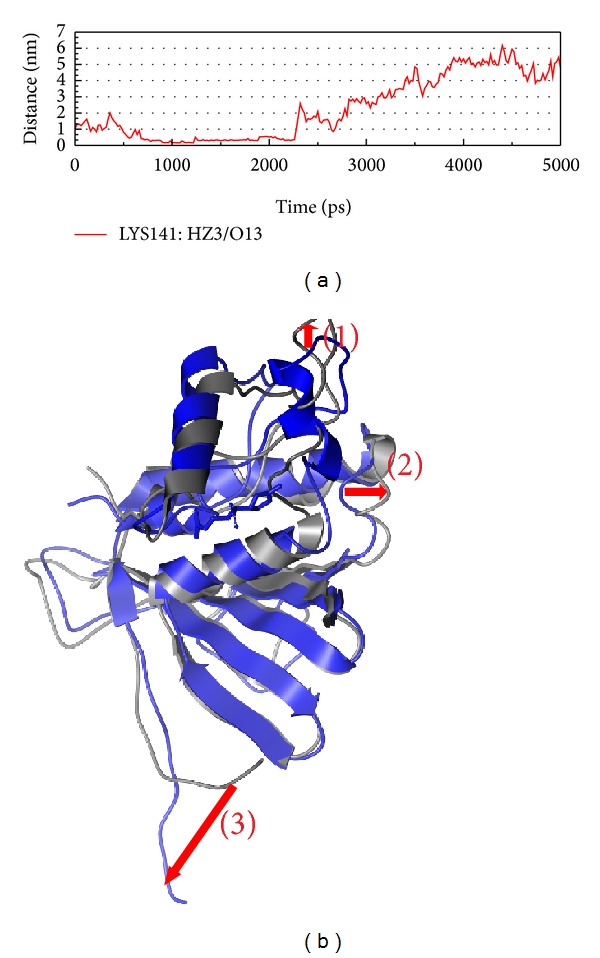
The variation of plantagoguanidinic acid and VIF complex in MD simulation. (a) H-bond variation, (b) structure variation. The (1)–(3) red color indicates the difference through MD.

**Figure 11:**
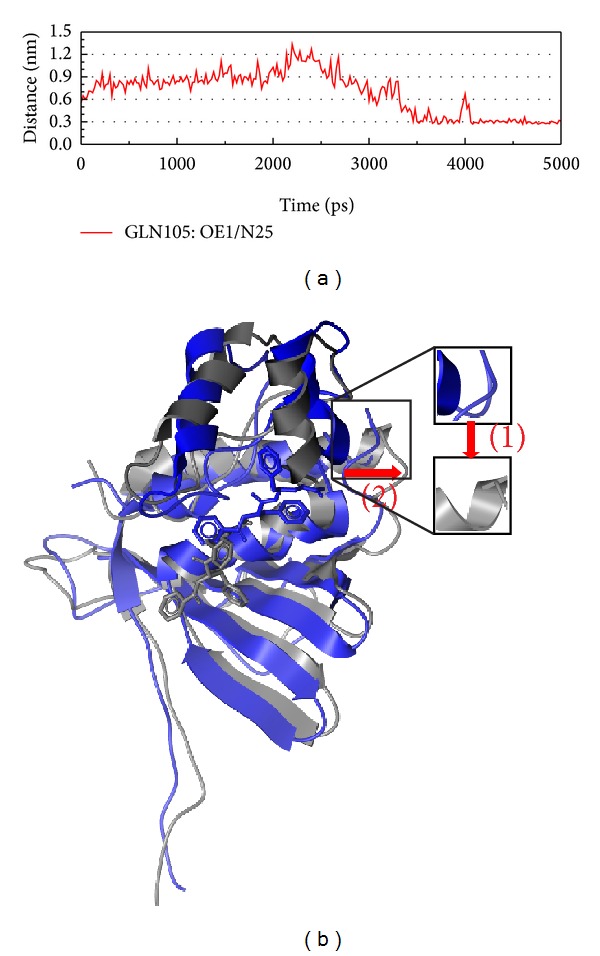
The variation of Aurantiamide acetate and VIF complex in MD simulation. (a) H-bond variation, (b) structure variation. The (1)-(2) red color indicates the difference through MD.

After the analysis of interaction, we should pay attention to the structure variation after the force of interaction (Figures 9, 10, and 11). In Figures 9(a) to 11(a), we could find that the H bond between protein and ligand is less. We suggest that the protein wants to prevent ligand target on the functional site; then the character of protein will make drug effect hardly. Besides Aurantiamide acetate, the structure variations of compounds interaction are only position variation; thus, we suggest that only the Aurantiamide acetate could have better efficacy for the inhibition of VIF.

**Figure 12:**
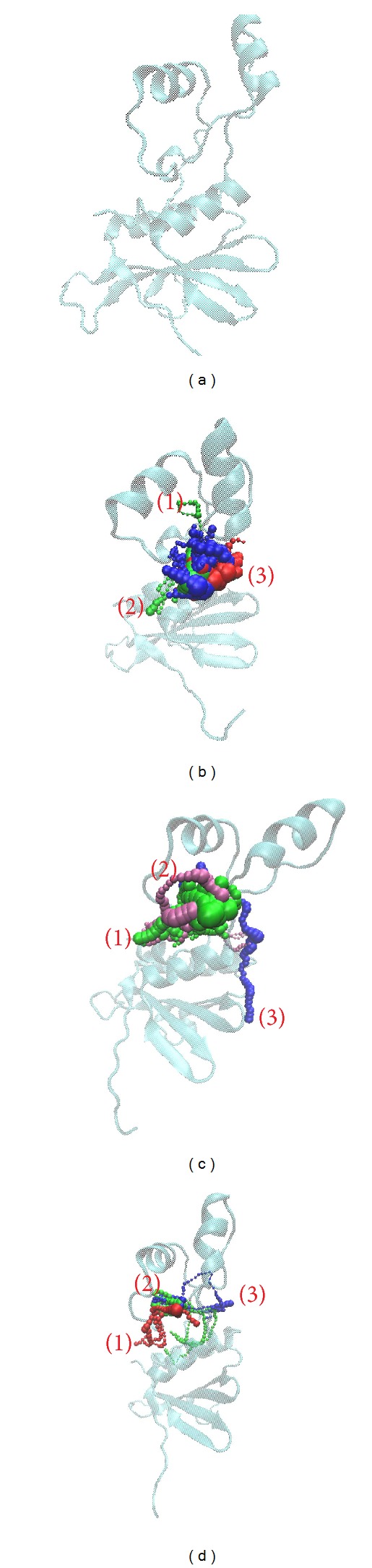
The pathway of VIF for compounds. (a) Apo VIF, (b) glutamic acid, (c) plantagoguanidinic acid, and (d) Aurantiamide acetate.

The pathway for ligand shows the path in protein (Figure 12). In this result, most of pathways are around the docking site, which indicates that the functional site might be focused on docking site and the protein has no other pole for the interaction.

## 4. Conclusion

In this study, the structure of computer-aided drug design is based on the theory of traditional Chinese medicine ligand screening compounds to inhibit the VIF. The compounds glutamic acid, plantagoguanidinic acid, and Aurantiamide acetate are selected from TCM database through several calculations to analyze the interaction and variation. Then, according to the RMSD, H bond interaction, and structure variation, we find the selected compounds move away from the docking site besides Aurantiamide acetate; this situation for drug design is not well. Thus, according to the simulation, we suggest that Aurantiamide acetate may the best compound to inhibit VIF and then help immunity to prevent HIV virus.

## Figures and Tables

**Table 1:** 

Name	Herb	Dock score	-PLP1	-PLP2
Glutamic acid	*Achyranthes bidentata Bl. *	175.136	32.52	29.59
Plantagoguanidinic acid	*Plantago asiatica L. *	91.191	47.61	44.23
Aurantiamide acetate	*Sargassum pallidum* (*Turn.*)/*Cordyceps sinensis* (*Berk.*) *Sacc.*/*Lycium chinense* Miller	82.182	70.96	66.45
